# Disparities Research at the Deep South Alzheimer’s Disease Center of the University of Alabama at Birmingham

**DOI:** 10.1093/geroni/igab046.379

**Published:** 2021-12-17

**Authors:** Maria Pisu, David Geldmacher

## Abstract

Residents of the US Deep South (Alabama, Georgia, Louisiana, Mississippi, and South Carolina) have a 20–30% higher risk of developing Alzheimer’s disease or related dementia (ADRD). Moreover, >20% of African Americans, who are at higher ADRD risk than whites, live in this region. Therefore, one important goals of the Deep South Alzheimer’s Disease Center (DS-ADC) of the University of Alabama at Birmingham is to spearhead research to address these disparities. This panel presents current DS-ADC research, with two presentations focusing on the local patient population and the last two on the Deep South population compared to the rest of the nation. Addressing the challenge of recruiting representative samples in clinical research, the first paper is part of a research program to understand difference that may exist between African American and white research participants. The second paper examines patients with multiple conditions, in particular dementia and cancer, showing a marked disadvantage in cognition outcomes for African Americans. The next two papers take a broader perspective to better understand the population of older adults with ADRD in the Deep South and in the rest of the US. The third paper examines socioeconomic and medical contexts of African American and white older Medicare beneficiaries with ADRD, and the fourth paper examines differences in utilization of specialists, ADRD drugs, and hospitalizations in the two regions taking these contexts into account. The discussant will close the session by placing these studies in the larger context of the disparities research at the DS-ADC.

